# Regulation of DNA replication-coupled histone gene expression

**DOI:** 10.18632/oncotarget.21887

**Published:** 2017-10-16

**Authors:** Qianyun Mei, Junhua Huang, Wanping Chen, Jie Tang, Chen Xu, Qi Yu, Ying Cheng, Lixin Ma, Xilan Yu, Shanshan Li

**Affiliations:** ^1^ Hubei Collaborative Innovation Center for Green Transformation of Bio-Resources, Hubei Key Laboratory of Industrial Biotechnology, College of Life Sciences, Hubei University, Wuhan, Hubei 430062, China; ^2^ Hubei Key Laboratory of Industrial Biotechnology, College of Life Sciences, Hubei University, Wuhan, Hubei 430062, China

**Keywords:** DNA replication, histone gene transcription, cell cycle

## Abstract

The expression of core histone genes is cell cycle regulated. Large amounts of histones are required to restore duplicated chromatin during S phase when DNA replication occurs. Over-expression and excess accumulation of histones outside S phase are toxic to cells and therefore cells need to restrict histone expression to S phase. Misregulation of histone gene expression leads to defects in cell cycle progression, genome stability, DNA damage response and transcriptional regulation. Here, we discussed the factors involved in histone gene regulation as well as the underlying mechanism. Understanding the histone regulation mechanism will shed lights on elucidating the side effects of certain cancer chemotherapeutic drugs and developing potential biomarkers for tumor cells.

## INTRODUCTION

In eukaryotes, DNA is tightly packaged in the nucleus in the form of chromatin. The fundamental structure unit of chromatin is nucleosome. Two copies each of the core histone proteins H2A, H2B, H3, and H4 associate with DNA to form the octamer, which is wrapped by ∼147 bp DNA about 1.75 rounds to form nucleosomes [1]. Such particles are formed at regular intervals and connected by 10-70 bp linker DNA. The linker histone H1 is required for compaction of nucleosomes into higher order chromatin structure. In addition, eukaryotes usually have several variants of histone H2A and H3, which have specialized and distinct functions.

During S phase, the chromatin structure is duplicated in coordination with DNA replication. Such process requires histone synthesis and their assembly into DNA to be efficiently coupled to DNA synthesis [2–4]. Based on their expression pattern, histone genes are generally classified into two classes [5, 6]. The first class comprises DNA replication tightly regulated histones whose expression level is high during DNA replication but reduced after DNA replication is completed. This class includes all four core histones (H2A, H2B, H3, H4) as well as the linker histone H1. At each cell cycle, doubling of the DNA material in S phase requires additional histones in order to maintain a proper DNA-histone ratio. However, constitutive expression of histones and accumulation of soluble histones outside S phase triggers chromosome aggregation or loss and are toxic to cells [7]. Thus, the synthesis and accumulation of these histones are tightly restricted to S phase to provide sufficient histones to assemble the replicated DNA into chromatin and prevent excess accumulation of histones at other cell cycle stages. For example, in thymidine- and aphidicolin-synchronized HeLa cells, there was a 15-fold increase in the level of histone mRNAs during S phase [8]. At the end of S phase or DNA synthesis is interrupted, cells turned off histone transcription and histone mRNA levels declined rapidly [8]. The second class of histones is composed of histone variants that are expressed at a relatively low level throughout the cell cycle, and are therefore regulated in a DNA replication-independent manner [6]. Maintaining a stable and balanced histone pool is of vital importance for appropriate gene regulation, cell cycle progression and genome stability. Here, we focused on discussing the DNA replication-dependent regulation of histone gene expression in yeast and mammals.

## HISTONE GENE TRANSCRIPTIONAL REGULATION

Eukaryotic cells usually contain multiple copies of core histone genes. In metazoans, there are 10-20 functional copies of these genes for each core histone proteins, which are clustered in two chromatin loci, *HIST1* and *HIST2*. The largest cluster, *HIST1*, comprising ∼80% histone genes is located on human chromosome 6 and mouse chromosome 13 [9]. The *HIST2* cluster containing the remaining 20% genes is located on human chromosome 1 and mouse chromosome 3 [10]. The repetitive nature and genomic clustering of these histone genes allow the formation of specialized subnuclear structures at these loci, called histone locus bodies (HLBs) or Cajal bodies, which are important features in the cell cycle control of histone gene expression in metazoans [11]. HLBs are enriched with transcription factors and 3’ processing components to facilitate co-regulation of histone gene expression [12]. The tandem repeat organization of these histone genes ensures each histone mRNA is equally produced [11].

**Figure 1:**
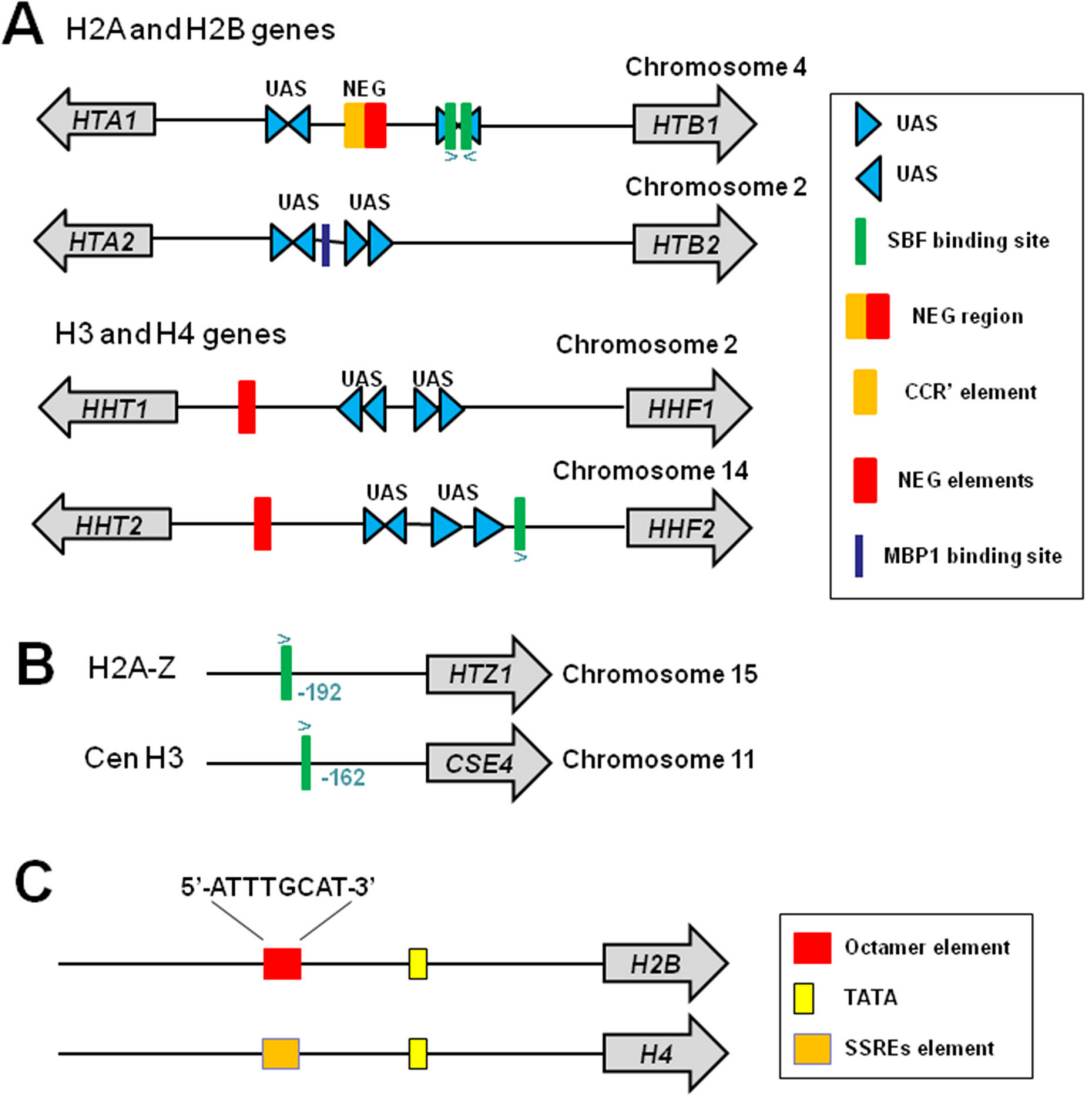
Histone gene structure in yeast and mammals **(A)** Structure of yeast canonical histone genes. All four core histone genes contain UAS elements. *HTA2* and *HTB2* have no NEG region. **(B)** Structure of yeast histone variants H2AZ and CenH3. **(C)** Structure of mammalian histone genes H2B and H4. Figures were adapted from [14].

In budding yeast, *Saccharomyces cerevisiae*, there are two copies for each core histone genes, which are arranged in an opposite orientation to the gene encoding its interaction partner within the nucleosome [5]: *HHT1*-*HHF1* and *HHT2*-*HHF2* encode H3-H4 pairs; *HTA1*-*HTB1*and *HTA2*-*HTB2* encode H2A-H2B pairs (Figure 1A). This divergent arrangement of histone promoters allows coordinated gene expression to get equal amount of all four core histones. The core histone gene promoters contain specialized DNA elements that enable cis-regulation of histone gene expression: UAS (upstream activating sequence) and NEG (Figure 1A).

### Cis-acting regulators

#### UAS (upstream activating sequence)

Histone UAS sequence has been found in the intergenic regions of yeast four core histone genes but not in the *HHO1*, *CSE4*, or *HTZ1* promoters [13] (Figure 1A and 1B). The four core histone pairs have four 16 bp UAS elements located in the middle regions of their promoters [14]. The UAS elements are responsible for S-phase-specific activation of histone genes and confer cell cycle-regulated transcription of a reporter gene [13]. The UAS elements are required for recruitment of transcription factors to activate gene transcription, including Spt10, which requires two histone UAS elements for high-affinity binding [15].

### NEG region

The histone core gene pairs *HTA1*-*HTB1*, *HHT1*-*HHF1* and *HHT2*-*HHF2* contain a specialized 54-bp NEG region required for histone gene repression [14]. Deletion of NEG region leads to constitutive expression of *HTA1* [13]. NEG region contains CCR (cell cycle control region) and NEG elements, which repress the expression of a reporter gene when inserted into a heterologous promoter [13]. Despite its established role in histone gene repression, it is unclear which transcriptional regulator(s) recognize and bind the NEG region. Histone gene pair *HTA2*-*HTB2* does not contain the NEG region.

In mammals, histone promoters also contain specialized cis elements that is required for histone gene expression (Figure 1C). Histone H2B promoter contains an octamer element (5’-ATTTGCAT-3’), which is bound by transcription activator Oct-1 (octamer-binding factor 1) [16]. Histone H4 promoter contains subtype-specific regulatory elements (SSREs), which is bound by the key transcription factor, HiNF-P (histone nuclear factor P) [17] (see below for more information).

#### Trans-acting regulators of histone gene expression

DNA replication-dependent histone gene expression is regulated by both positive and negative factors (Table 1). In the following sections, we will describe these regulators in detail.

**Table 1:** Summary of trans-acting regulators in histone gene expression in budding yeast

Regulators	Roles in histone gene expression	References
**Transcriptional repressors**		
HIR	The HIR complex localizes to the NEG region of histone genes to repress histone expression through its histone chaperone activity to assemble nucleosomes that block the recruitment of transcription machinery.	[5, 19, 20]
Asf1	Asf1 primarily functions to repress histone gene expression outside S phase.	[5]
Rtt106	Rtt106 is required for recruitment of the RSC (remodels structure of chromatin) chromatin remodelling complex to NEG-containing chromatin outside S phase. Rtt106 recruits SWI/SNF to activate gene transcription at S phase.	[19, 32, 33]
RSC complex	RSC is recruited by Rtt106 to repress histone gene expression.	[8, 14, 34]
**Transcriptional activators**		
Spt10	Spt10 binds specifically to UAS elements within histone promoters to activate histone gene transcription. Spt10 inhibits gene expression outside S phase through recruiting HIR and associated proteins at histone gene promoters.	[37]
Spt21	Spt21 serves as a master regulator in S-phase-dependent histone gene expression. Spt21 is required for Gcn5 binding and probably histone acetylation at *HTA2*-*HTB2* promoters, which subsequently activates histone gene expression. Spt21 is degraded by APC/C^cdh1^ at G1 phase to ensure that histone gene expression is tightly restricted to S phase. Spt21 inhibits gene expression outside S phase.	[6, 41]
SBF	SBF binds to UAS elements of histone promoters and its binding sites overlap and are mutually exclusive with Spt10 binding sites. SBF is responsible for an early peak of histone transcription in late G1 phase.	[50, 51]
MBF	MBF is required for histone gene activation.	[50]
Yta7	Yta7 functions as a boundary element to activate histone gene expression during early G1, G2, M and early S phases. In mid S phase, Yta7 is heavily phosphorylated by Cdk1 and CK2, which is required for Yta7 dissociation from histone gene promoters and effective elongation of RNA polymerase II along histone genes.	[36]
Rtt109 /Vps75	Rtt109 enhances the transcription of the *HTA1* gene by facilitating chromatin disassembly at the locus through deposition of H3K56ac-H4 dimers.	[62, 63]
SWI/SNF	SWI/SNF complex activates histone expression perhaps by evicting nucleosomes at the histone promoters to expose the UAS elements for Spt10 and SBF	[27]

### Transcription repressors

#### HIR (histone regulatory complex)

*HIR1*, *HIR2*, and *HIR3* were identified as negative regulators of histone transcription in the genetic screen [18, 19]. Hir1, Hir2, Hir3 form a stable repressor complex called the histone regulatory (HIR) with Hpc2 [20]. In synchronized cells, the HIR complex primarily represses histone gene expression outside S phase [19]. When Hir1 and Hir2 are artificially tethered to yeast promoters, they repressed gene transcription [21]. The phenotype of *HIR*/*HPC* mutant is similar to that of the *NEG* deletion mutant and HIR complex represses the expression of three histone gene pairs with the exception of *HTA2*-*HTB2*, which has no NEG elements [5, 18]. ChIP (chromatin immunoprecipitation) analysis showed that all four HIR complex subunits specifically localize to the NEG region of *HTA1*–*HTB1* [19]. All these data indicate that HIR complex functions through the NEG elements and the binding of HIR complex to the NEG region is required to inhibit the expression of three of the four histone loci [5]. The repressive nature of the HIR complex in histone gene expression depends on its histone chaperone activity to assemble nucleosomes that block the recruitment of transcription machinery [20]. Although the HIR complex possesses DNA binding activity, HIR shows no selectivity towards NEG and non-NEG containing DNA, suggesting that additional sequence-specific DNA-binding factor(s) is required for HIR recruitment to the CCR/NEG region [22]. Deletion of *SPT10* or *SPT21* reduced binding of Hir1 at NEG region of *HTA1*-*HTB1* locus [6], making it possible that Spt10 and Spt21 are required for HIR complex recruitment. It is unknown whether Spt10/Spt21 directly recruit the HIR repressor complex and whether there is additional protein factor(s) to recruit HIR.

The functions of yeast HIR complex are conserved among eukaryotes. The typical example is HIRA (Histone Regulator A), the human homolog of Hir1 and Hir2 [5]. Ectopic expression of HIRA represses transcription of histone genes and triggers a concerted block of DNA synthesis [23]. The recruitment of HIRA requires histone H2B to be phosphorylated at tyrosine 7 upstream of the *HIST1* gene cluster in human cells by WEE1 kinase [24]. Mutation of the corresponding tyrosine in H2B or deletion of WEE1-related cell cycle kinase, *SWE1* in budding yeast derepresses the expression of all four core histone gene pairs [19]. Besides HIRA, the roles of other putative HIR members in histone gene regulation remain unclear, i.e. UBN1, the homolog of Hpc2; CABIN1, the homolog of Hir3 [5]. Future efforts are required to address this question.

### Asf1

HIR repressor complex requires H3-H4 histone chaperones, Asf1 and Rtt106 to repress gene expression [5]. Deletion of *ASF1* did not show an apparent defect in repressing histone gene expression in asychronized cells [19, 25]; however, in α-factor synchronized cells, deletion of *ASF1* results in derepression of histone expression in G1, G2, and M phases [25], indicating Asf1 primarily functions to repress histone gene expression outside S phase. Asf1 is recruited to HIR-dependent NEG region via a direct interaction between Asf1 and Hir1 [25–27].

#### Rtt106 (regulator of Ty1 transposition)

*RTT106* was originally identified as a gene whose deletion increases the transposition of the Ty1 retroelement [28]. Subsequent genetic analysis revealed that Rtt106 is functionally related to Asf1 and Hir1 [29]. The role of Rtt106 in histone gene repression was revealed by a dual reporter-based synthetic genetic array screen [19]. Rtt106 is specifically recruited to NEG region depending on HIR and Asf1 [19]. Further in-depth research showed that HIR and Asf1 recruit Rtt106 through H3-H4 tetramers as mutations that reduce the histone-binding ability of Rtt106 reduced its recruitment [27, 30]. Rtt106 homo-oligomerization is required for the binding of Rtt106 to H3-H4 tetramers [31]; therefore, it is possible that Rtt106 homo-oligomerization could mediate histone gene expression. Deletion of *ASF1* or *RTT106* causes a nucleosome-free region in three histone gene pairs promoters, similar to *HIR* deletion mutants [19]. Moreover, Rtt106 is required for recruitment of the RSC (remodels structure of chromatin) chromatin remodeling complex to NEG-containing chromatin outside of S phase [32]. These data suggest that cell-cycle repression of histone genes depends on a nucleosome assembly pathway mediated by Asf1, HIR complex, Rtt106 and RSC [33]. Nonetheless, Rtt106 has a dual role in histone gene expression as it also recruits SWI/SNF to activate gene transcription in S phase [32]. How Rtt106 coordinates the recruitment of negative regulator, RSC versus positive regulator, SWI/SNF to histone genes remains to be determined.

#### RSC (remodels structure of chromatin) complex

One ATP-dependent chromatin remodeling complex recruited by Rtt106 to histone gene promoters is RSC complex. Rtt106 is required for the recruitment of RSC to the HIR-dependent histone genes [8], presumably via a physical interaction between RSC and Rtt106 [32]. The RSC complex is also recruited to the *HTA1*-*HTB1* locus in a HIR-dependent manner [32]. A genome-wide analysis of RSC localization revealed HIR-dependent RSC association with CCR/NEG-containing chromatin regions [30]. RSC recruitment to histone loci coincides with the periods of histone gene repression [30]. Specifically, the maximal binding of RSC to histone promoters is outside of S phase when histone genes are repressed [14, 34], implying that RSC is functionally related to histone gene repression. RSC along with HIR/Asf1/Rtt106 histone chaperones assemble nucleosomes at histone promoters at the end of S phase, which occludes the recruitment of general transcription factors and/or RNA polymerase II. However, in *RSC* mutations, we and other labs found that histone gene transcription was not significantly affected [35]. Whether NEG-regulated histone gene repression depends on the activity of RSC complex remains to be tested. It is likely that synchronized cells are required to observe any changes of histone gene expression in *RSC* mutants.

**Figure 2:**
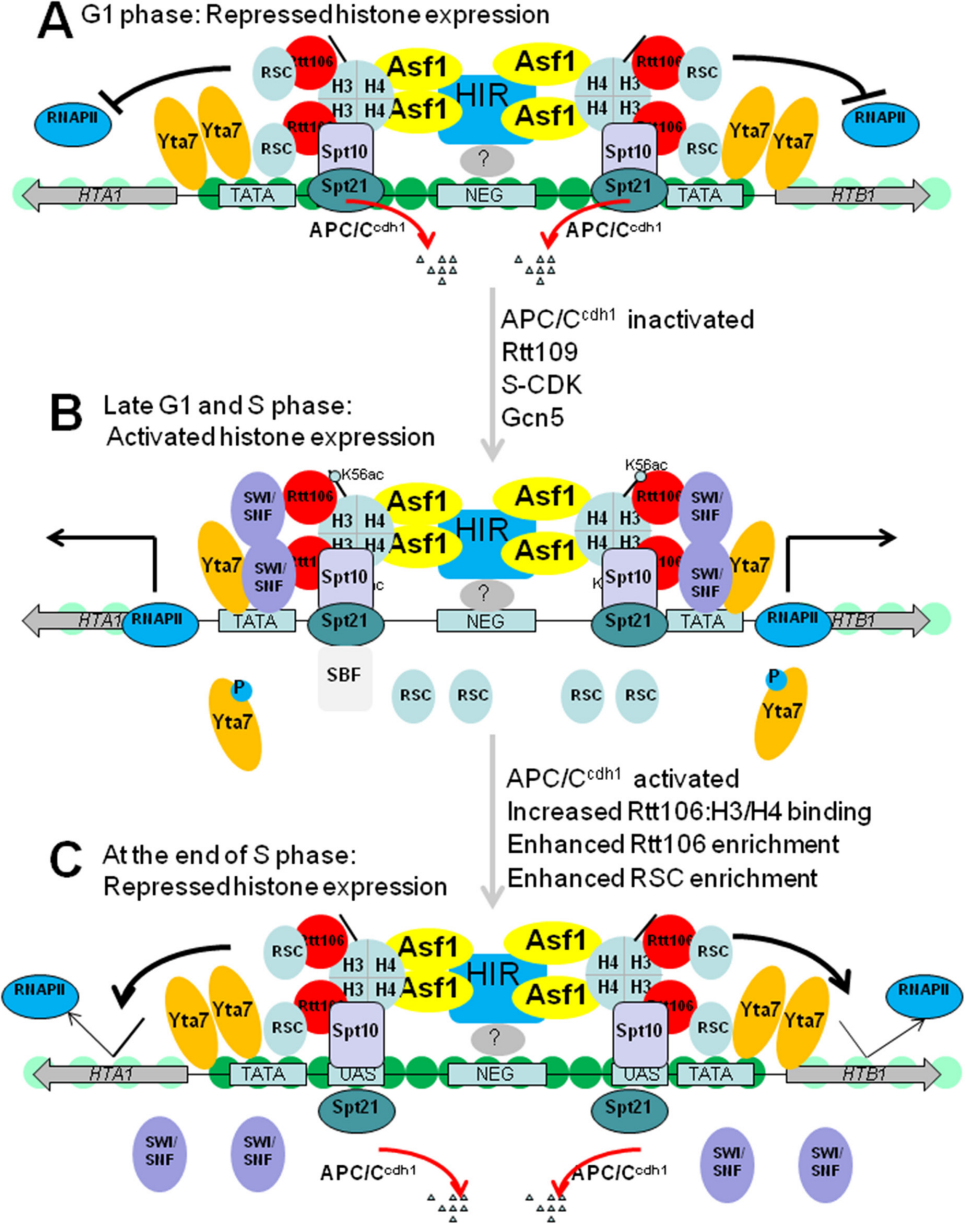
Model for histone gene regulation in budding yeast **(A)** Repressed histone gene transcription in G1 phase. HIR is recruited to the NEG region by a yet unknown factor(s), which then recruits Asf1, H3-H4 tetramers and Rtt106. RSC is recruited by Rtt106 and functions along with HIR/Asf1/Rtt106 to form a repressive chromatin structure to occlude the basal transcription machinery. APC/C^cdh1^ degrades Spt21 at G1 phase to ensure that histone gene expression is tightly restricted to S phase. **(B)** Activated histone gene transcription in late G1 and S phase. Spt10 and Spt21 are recruited to UAS region to enhance the binding of Gcn5 and other HATs to acetylate histones in histone promoters. Rtt109-dependent incorporation of H3 acetylated at K56 (H3K56ac) enables recruitment of SWI/SNF and probably dissociation of RSC complex, which removes nucleosomes in the promoter, facilitating the recruitment of RNA polymerase II. S phase forms of CDK1 (S-CDK) then phosphorylate Yta7 causing its eviction from promoters, which is important for efficient promoter escape and transcription elongation by RNA polymerase II. **(C)** Negative feedback repressed histone gene transcription at the end of S phase. At the end of DNA replication, the chaperones are fully charged with histones and inhibit gene transcription. Figures were adapted from [5]. RSC, remodels structure of chromatin;RNAPII, RNA polymerase II; S-CDK, S phase forms of CDK1.

How are histone genes repressed by the above regulators? A simple model explaining repression of NEG-dependent core histone genes outside of S phase in budding yeast is illustrated in Figure 2A. When cells are in a cell cycle outside of S phase, the NEG element at the center of divergent promoters is recognized by an unknown factor(s), which recruit HIR complex. HIR complex subsequently recruits histone chaperone Asf1 through a direct interaction. HIR and Asf1 recruit Rtt106 through H3-H4 tetramers. Rtt106 collaborates with RSC, along with HIR complex to assemble histone H3 and H4 into chromatin, which sequesters the promoter sequences and prevents the recruitment of the general transcription apparatus and RNA polymerase II [36].

### Transcription activators

#### Spt (suppressor of Ty) proteins: Spt10 and Spt21

Spt10 and Spt21 were originally identified as suppressors of the transcriptional defect caused by Ty1 transposon insertion. The mutations in these two genes enable the 3’ LTR (long terminal repeat) of Ty1 elements inserted at various positions of the yeast genome to function as the promoter to initiate gene transcription [33]. As described above, Spt10 contains a DNA-binding domain that binds specifically to UAS elements within histone promoters [15, 37]. Spt10 contains a dimerization domain in its N terminal domain, contributing to its cooperative binding to UAS elements. Spt10 contains a putative histone acetyltransferase (HAT) domain that is required for histone gene activation but there is no direct evidence to demonstrate its HAT activity due to lack of a stable catalytic dead mutant [38]. Spt10 is required for S-phase-dependent histone acetylation in the regulatory region of all four core histones, including H3K56ac [39], which could be due to its indirect effect on the recruitment of H3K56 acetyltransferase, Rtt109. Further ChIP analysis is required to explore this possibility. Another possibility is that Spt10 could possess the KAT (lysine acetyltransferase) activity like Gcn5 that allows it to acetylate non-histone proteins to modulate their functions [40]. Further efforts are required to explore this possibility. Spt10 is recruited to histone promoters in a Spt21- and cell cycle-dependent manner [38]. Interestingly, Spt10 recruits and stabilizes the S-phase-specific Spt21 at both *HTA1*-*HTB1* and *HTA2*-*HTB2* promoters via the physical interaction between Spt10 and Spt21 [6].

Spt21 is a cell cycle oscillator that serves as a master regulator in S-phase-dependent histone gene expression [6]. Spt21 is required for Spt10 binding and histone gene activation during S phase [6]. In *SPT21* deletion mutant, the transcription of histone genes *HTA2*, *HTB2*, and *HHF2* was reduced [41]. Spt21 is required for Gcn5 binding and probably histone acetylation at *HTA2*-*HTB2* promoters, which subsequently activates histone gene expression [6]. It should be noted that deletion of *GCN5* leads to minor defect in histone gene expression [6], implying that other histone acetyltransferases play a redundant role in histone gene activation. Acetylated histones alleviate HIR (histone regulatory complex)-dependent repression probably by facilitating the recruitment of ATP-dependent chromatin remodeling complex SWI/SNF [6] (see below).

The protein levels of Spt21 are cell cycle regulated with a peak in S phase, consistent with its oscillator role in histone gene regulation [6]. Spt21 contains a canonical KEN box, which is a substrate recognition motif for anaphase-promoting complex/Cdh1 (APC/C^cdh1^), a G1-specific E3 ubiquitin ligase composed of 13 distinct core proteins [6]. APC/C^cdh1^ degrades Spt21 at G1 phase to ensure that histone gene expression is tightly restricted to S phase [6]. However, it is unknown how Spt21 is degraded in G2 and M phases. Spt21 has a homolog in fission yeast, Ams2, which couples histone expression with DNA replication and is degraded by APC/C^cdh1^ ubiquitin ligase in G1 and SCF^pof3^ ubiquitin ligase in G2 and M phases [42, 43]. It will be interesting to examine whether the F-box protein Dia2, the homolog of SCF^pof3^in budding yeast, degrades Spt21 in G2 and M phases.

The homolog of Spt21 in mammals is NPAT (Nuclear Protein Ataxia-Telangiectasia Locus), whose expression oscillates with cell cycle with a peak at S phase [44]. NPAT is the main factor responsible for the S phase-specific transcriptional activation of histone genes at both *HIST1* and *HIST2* clusters [44, 45]. NPAT is also essential for DNA replication-dependent histone mRNA 3’ processing by recruiting cyclin-dependent kinase-9 (CDK9) and increasing histone H2B monoubiquitination (H2Bub1) at histone gene promoters [46, 47]. NPAT is phosphorylated by cyclin E/Cdk2 to trigger a cascade of protein recruitments to the HLBs at the G1/S transition phase to activate histone gene transcription [12]. Similar to Spt21, NPAT also contains a putative KEN box despite little is known about its function. It seems the functions of Spt21 are highly conserved from yeast to metazoans.

It should be noted that Spt10 and Spt21 restrict histone synthesis to S phase not only by activating S-phase-specific gene expression, but also by inhibiting gene expression outside S phase through recruiting HIR and associated proteins at histone gene promoters [6]. Deletion of *SPT21* caused a derepression of *HTA2* during G2 and M phases [6].

#### SBF (SCB-binding factor) and MBF (MCB-binding factor)

SBF and MBF are DNA-binding transcription factors primarily required for activation of gene expression at the G1/S phase [48]. Genome-wide ChIP analysis revealed that SBF and MBF could be involved in histone gene regulation in budding yeast [48]. A MBF binding motif has been found in front of most fission yeast histone genes [49], implying that MBF may play a role in modulating histone expression in fission yeast. SBF is a heterodimer of Swi4 and Swi6, and MBF is a heterodimer of Mbpl and Swi6. Swi4 and Mbp1 are DNA-binding factors in SBF and MBF, respectively [48]. Deletion of *SWI4*, *MBP1*, or *SWI6* reduced the expression of histone genes, indicating that MBF and SBF play an important role in histone gene activation [50]. The binding sites of Swi4 and Mbp1 are within the histone UAS pairs, which overlap and are mutually exclusive with Spt10 binding sites [51]. Mutation of Spt10 is synthetic lethal when combined with deletion of either *SBF* or *MBF*, implying that Spt10 and SBF/MBF play a distinct role in histone gene activation [5, 50]. Indeed, subsequent studies showed that SBF is responsible for a small early peak of transcription in late G1 phase, while Spt10 contributes a later, much larger peak in S phase [51]. This different activation timing by Spt10 and SBF suggests that their functions are not redundant [51]. However, it remains unclear about the mechanism by which SBF and MBF activate histone gene transcription. SBF has been shown to be required for cell cycle-regulated histone modifications, i.e. H3K79 dimethylation, which is enriched in M/G1 phase-regulated genes as well as SBF-bound promoters [52]. It is plausible that SBF activates cell cycle-dependent histone gene expression via H3K79 dimethylation.

### Yta7

The Yeast tat-binding analog 7 (Yta7) protein was functionally related to Rtt106-dependent histone gene regulation [53]. Yta7 was characterized as a protein with chromatin barrier function to separate silent and active chromatin around the silent mating locus, *HMR* [54, 55]. Yta7 prevents the spreading of silent chromatin from the *HMR* locus to ensure its neighboring genes unaffected [56]. Yta7 was involved in histone gene activation by the following evidence: deletion of *YTA7* causes a severe growth phenotype when combined with *HIR1* or *ASF1* deletion [53]; Yta7 specifically localizes to regulatory regions of the HIR-regulated histone loci [19, 27, 53]; histone transcriptional levels are reduced in *YTA7* deleted cells [19, 27]. Yta7 is required for histone gene activation by preventing the spreading of repressive Rtt106 from the NEG region, thereby restricting HIR- and Asf1-recruited Rtt106 to the regulatory region of NEG-regulated histone gene pairs, facilitating the recruitment of RNA polymerase II [19, 27]. Deletion of *YTA7* leads to mis-localization of Rtt106, which spreads from the NEG region through the *HTA1* ORF [19, 27]. Spreading of Rtt106 into the *HTA1* coding region could repress gene transcription by interfering with recruitment of RNA polymerase II [19]. The binding of RSC at the NEG region is restrained by Yta7 as deletion of *YTA7* caused the spreading of RSC into the coding region [27]. In synchronized cells, Yta7 acts as a boundary element during early G1, G2, M and early S phases [36]. In mid S phase, Yta7 is heavily phosphorylated by cyclin-dependent kinase 1 (Cdk1) and casein kinase 2 (CK2), which is required for Yta7 dissociation from histone gene promoters and effective elongation of RNA polymerase II along histone genes [36].

Yta7 possesses an AAA-ATPase domain and a noncanonical bromodomain. Of these two domains, only the Yta7 AAA-ATPase domain is required to prevent Rtt106 spreading, since a *YTA7*-*K460A* point mutation which is predicted to compromise its AAA-ATPase activity is unable to prevent Rtt106 spreading [36]. Subsequent experiments showed that Yta7 AAA-ATPase domain is required for correct position of Rtt106 and RSC, RNA polymerase II recruitment and proper core histone gene expression [36]. The Yta7 bromodomain showed no preference for acetylated histones, but it is still required for specific binding and barrier activity of Yta7 on *HMR* locus [54]. It is likely that Yta7 bromodomain contributes to Yta7 recruitment by binding to acetylated non-histone proteins. Nevertheless, deletion of Yta7 bromodomain did not affect *HTB1* transcription [54], indicating that Yta7 bromodomain is not required for Yta7 binding and boundary activity on histone loci.

Yta7 is conserved across evolution like other histone gene regulators [5]. The human homolog of Yta7 is ATAD2, which contains a bromodomain and an ATPase domain and its expression levels correlate with the clinical outcome of breast cancer patients [57]. ATAD2 controls chromatin dynamics and genome transcriptional activities [58]. Moreover, ATAD2 is phosphorylated at CK (casein kinase) and CDK (cyclin-dependent kinase) consensus sites [36]. All these data implied that like Yta7, ATAD2 could function in DNA replication-coupled histone synthesis. Further efforts are required to address this question to shed light on the functions of ATAD2 in tumorigenesis.

#### Rtt109 and Vps75

Rtt109 and Vps75 were identified as histone gene activators in a genome-wide screen [19]. Expression of *HTA1* is decreased in *RTT109* deleted cells, indicating its positive role in histone gene expression [33]. *RTT109* encodes a HAT that specifically acetylates H3K56 (H3K56ac), a modification associated with chromatin assembly [59, 60]. During S phase, Rtt109 enhances the transcription of *HTA1* gene (and likely other histone genes) by facilitating chromatin disassembly at the locus through deposition of H3K56ac-H4 dimers [61, 62]. Rtt109 forms a stable protein complex with Vps75 which is also a H3-H4 histone chaperone [63]. Rtt109-Vps75 also acetylates H3K9 in gene coding regions with the help of Asf1 to prevent the cryptic transcription [64, 65]. It is unknown about the precise function of Vps75 in histone gene expression.

#### SWI/SNF

The SWI/SNF is an ATP-dependent chromatin remodeling complex that is required for the expression of histone *HTA1*-*HTB1* locus [66]. Deletion of *SNF5* reduces the expression of core histones [39, 66]. SWI/SNF complex activates histone expression perhaps by evicting nucleosomes at the histone promoters to expose the UAS elements for Spt10 and SBF [27]. SWI/SNF subunits Swi2 and Snf5 are specifically recruited to the NEG region with the assistance of histone chaperone Rtt106 [32]. Rtt106 recruits SWI/SNF via a physical interaction [32]. Rtt106-dependent SWI/SNF recruitment to these histone loci is cell cycle regulated and restricted to late G1 phase just before the peak of histone gene expression in S phase [32]. In addition, Spt10 and H3K56ac are required for recruitment of SWI/SNF to *HTA1* locus since deletion of *SPT10* or mutation of H3K56R reduces Snf5 recruitment at histone loci [67]. The recruitment of SWI/SNF complex has not been examined in *RTT109* mutant by ChIP, hence it is not clear whether it is H3K56 residue or H3K56ac that is required for SWI/SNF recruitment. As Rtt109-catalyzed H3K56ac is indispensable for Rtt106 binding to histone loci [27], it is conceivable that Rtt109 contributes to the recruitment of SWI/SNF indirectly via H3K56ac and Rtt106. Interestingly, HIR subunits are also required for recruitment of SWI/SNF to histone genes [66].

The model describing the activation of S phase-specific transcription of NEG-regulated histone genes in budding yeast is depicted in Figure 2B. The S phase-activated transcription of histone genes occurs by overcoming HIR/Rtt106-mediated repressive chromatin [19]. During G1 phase, the APC/C^cdh1^ prevents Spt21 from accumulation and premature transcription of histone genes. During S phase, when APC/C^cdh1^ is inactivated, Spt21 accumulates and is recruited to all histone gene promoters by Spt10. One downstream effector of Spt10/Spt21 is Gcn5, which acetylates histone H3 and H4 within histone gene promoters [44]. Another downstream effector is SWI/SNF. SWI/SNF is recruited to the NEG-regulated histone loci by Rtt106 through a physical interaction. At late G1 or early S phase, Yta7 functions as a boundary protein to prevent the spread of Rtt106 and RSC from NEG region into histone promoters, facilitating the recruitment of RNA polymerase II. After that, Yta7 is heavily phosphorylated by Cdk1 and CK2 to promote efficient RNA polymerase II elongation [36].

What is the signal to stop histone transcription at the end of the S phase? The negative feedback model proposes that soluble histone levels are monitored by HIR/Asf1/Rtt106 chaperone complex [5, 14, 27] (Figure 2C). When DNA replication is almost finished, there are fewer available genomic locations for assembly of newly synthesized histones and thus HIR, Asf1, and Rtt106 become fully charged with their histone substrates [5, 14, 27]. The fully charged histone chaperones facilitates the repressive chromatin assembly at the NEG region, leading to concomitant promoter occlusion and reduced recruitment of RNA polymerase II [5, 14, 27].

#### Histone post-translational modifications

Histone expression is regulated by histone modifications. As described above, histone acetyltransferase Gcn5 positively regulates histone gene expression [6, 68]. Gcn5 is recruited to histone gene promoters by Spt21 and Spt10 to acetylate histones at core histone gene promoters [6]. *GCN5* deletion cells had reduced histone levels compared to the parental wild type cells [68]. Histone H4 acetylation by Tip60 histone acetyltransferase complex is required for histone activation in mammals [69]. The acetylated histones activate histone gene expression probably by altering the nucleosome structure and facilitating the recruitment of bromodomain-containing transcription factors, such as SWI/SNF, SAGA (Spt-Ada-Gcn5 acetyltransferase), etc [70–72].

Histone gene expression is inhibited by WEE1 kinase-catalyzed histone H2B phosphorylation at tyrosine 37 (H2BY37) and this mechanism is conserved from yeast to mammals [24]. Loss of expression or inhibition of WEE1 kinase abrogates H2BY37 phosphorylation and increases histone transcription in yeast and mammalian cells [24]. H2BY37 phosphorylation reduces the binding of NPAT and RNA polymerase II but increases the recruitment of the histone chaperone HIRA upstream of the *HIST1* cluster [24].

Cdk9 (Cyclin-dependent kinase 9) is essential for replication-dependent histone mRNA 3’ processing by maintaining the global and gene-associated levels of histone H2B monoubiquitination (H2Bub1) [46]. The recruitment of Cdk9 to histone genes increases Ser2 phosphorylation of RNA polymerase II CTD (C-terminal domain) that is essential for the binding of PAF (Polymerase II Associated Factor), which then stimulates the activity of the E3 ubiquitin ligase complex RNF20/40 to catalyze H2Bub1 [73]. On the histone loci, H2Bub1 levels are specifically elevated near the 3’ cleavage sites [46]. H2Bub1 can recruit ASH2L or SET2D methyltransferase complex that methylates histone H3K4 [74]. This modification serves as a docking site for CHD1 (Chromodomain helicase DNA binding protein 1), which in turn recruits spliceosomal components, in particular the U2 snRNP [75, 76]. Furthermore, one component of the PAF complex, the tumor suppressor Cdc73, has been shown to associate with CPSF and CstF and contribute to the 3’ maturation of polyadenylated histone mRNAs [22, 77].

### Other histone gene regulators

Durano et al. reported that Nhp6 proteins, the yeast homolog of HMGB1, repress histone gene expression as histone genes were up-regulated in *NHP6* mutant [78]. Nhp6 proteins are encoded by *NHP6A* and *NHP6B* and regulate gene transcription by stimulating the formation of the TBP-TFIIA-DNA complex or acting as a part of the histone chaperone FACT (FAcilitates Chromatin Transcription) complex [78]. Nhp6 is localized in proximity of the transcription start site of the histone gene clusters to stabilize nucleosomes in the promoter region [78]. In addition, the over-expressed histones in the *NHP6* mutant is accompanied by down-regulated translation efficiency [78]. But it is unknown how Nhp6 is recruited to histone gene loci. Identification of Nhp6 interaction proteins by yeast two-hybrid assay and mass spectrometry should give us some clues.

### Transcription regulation of histone genes in mammals

**Figure 3:**
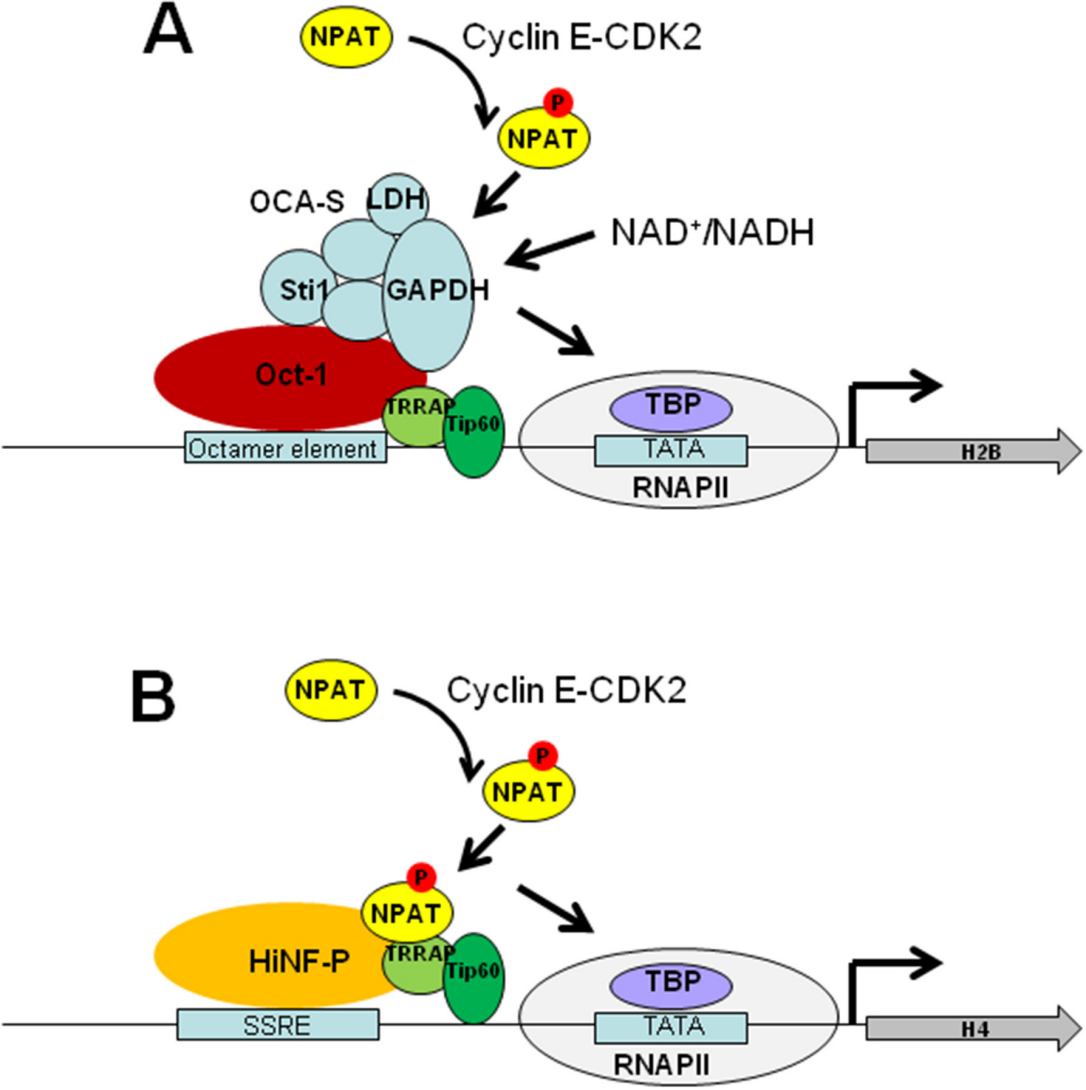
Model for histone gene activation in mammals **(A)** Activation of histone H2B. Oct-1 binds to octamer elements in H2B promoter. During S phase, activated cyclin E/CDK2 complex phosphorylates NPAT. In combination with NPAT, Oct-1 recruits OCA-S to H2B promoter to activates the expression of H2B. The NAD^+^/NADH directly controls the transcription of the H2B gene via regulating the interaction between Oct-1 and OCA-S. **(B)** Activation of histone H4. HiNF-P binds to SSRE within H4 promoters and recruits NAPT and RNA polymerase II to activate gene transcription. NPAT recruits the Tip60 histone acetyltransferase complex to acetylate histone H4 at the G1/S-phase transition. At the end of S phase, the tyrosine kinase WEE1 is recruited to histone promoters to phosphorylate H2B tyrosine 37, which evicts NPAT and RNA polymerase II and instead recruits HIRA to repress histone gene expression. Figures were adapted from [83]. NPAT, Nuclear Protein Ataxia-Telangiectasia Locus; RNAPII, RNA polymerase II; TRRAP, transformation/transactivation domain-associated protein; SSRE, subtype-specific regulatory elements; OCA-S, Oct-1 co-activator in S-phase; HiNF-P, histone nuclear factor P; TBP, TATA-box binding protein.

Unlike histone gene expression in yeast, the canonical histones in mammals are constitutively transcribed by RNA polymerase II with their rate of transcription increases remarkably during S phase [79] (Figure 3). NPAT is constitutively present throughout the cell cycle in Cajal bodies and is required to stimulate histone gene transcription and cell entry into S phase [80]. At the beginning of S phase, cyclin E-CDK2 (cyclin dependent kinase 2) phosphorylates NPAT in these bodies, and the phosphorylated form of NPAT persists throughout S phase, resulting in elevated expression of canonical histone genes [81]. Histone H2B promoter contains an octamer element, which is bound by transcription activator Oct-1 (Figure 3A). Oct-1 activates S-phase-specific H2B transcription by recruiting OCA-S (Oct-1 co-activator in S-phase), a co-activator complex comprising the glycolytic enzymes GADPH (glyceraldehyde-3-phosphate dehydrogenase) and LDH (lactate dehydrogenase) as well as other subunits [16]. Oct-1 binds the essential octamer site in the H2B promoter throughout the interphase; however, OCA-S occupies the H2B promoter only in the S phase [16]. The interaction between Oct-1 and OCA-S is controlled by intracellular NAD^+^/NADH [82]. Cyclin E-CDK2-phosphorylated NPAT could facilitate the binding and/or activation of OCA-S complex in S phase [83]. Histone H4 promoter contains subtype-specific regulatory elements (SSREs), which is bound by the key transcription factor, HiNF-P (histone nuclear factor P) [17] (Figure 3B). HiNF-P is required for recruitment of NAPT and RNA polymerase II to H4 promoters to activate gene transcription [17]. NPAT has been reported to interact with transformation/transactivation domain-associated protein (TRRAP) and Tip60, two components of the Tip60 histone acetyltransferase complex [69]. Similar to Gcn5 in budding yeast, TRRAP/Tip60 are recruited to histone gene promoters to acetylate histone H4 at the G1/S-phase transition in a NPAT-dependent manner [69]. Suppression of TRRAP or Tip60 expression abrogates histone gene activation [69]. At the end of S phase, the tyrosine kinase WEE1 is recruited to histone promoters to phosphorylate H2B tyrosine 37, which evicts NPAT and RNA polymerase II and instead recruits HIRA to repress histone gene expression [24].

## POST-TRANSCRIPTIONAL REGULATION OF HISTONE GENES

In multicellular metazoans, the elevated histone mRNAs at S phase also require histone mRNA to be properly processed. Histone genes do not contain introns and their mRNA 3’ ends are produced by cleavage of longer precursors [12, 84]. Their mRNAs contain a highly conserved stem-loop structure within the 3’ untranslated region (UTR) of the mRNA that is crucial for their regulation [85]. This novel 3’ structure requires a distinct set of factors for histone mRNA. The critical factor in this process is stem-loop binding protein (SLBP), which binds to this stem-loop structure and regulates mRNA processing [86]. SLBP and U7 snRNP, which is a component of the U7 small nuclear ribonucleoprotein, contribute to mRNA 3’ processing by recruiting the cleavage factors including CPSF73 (cleavage and polyadenylation specificity factor 73), CPSF100, symplekin and FIP1 [87]. This unique mode of RNA processing is important to ensure that adequate amounts of histone proteins are produced to pack the newly synthesized DNA during S phase and that the expression of these genes is low in other cell cycle phases [12]. At the end of S phase, the half-life of these histone mRNAs drops dramatically in response to destabilization of the 3’ ends. The 3’ stem-loop and SLBP are required for mRNA degradation by recruiting the proteins necessary to add a short oligo(U) tail to histone mRNAs that is being translated [88]. The La protein, which contains the RNA binding La motif, has been reported to stabilize S phase histone mRNAs and promote their translation [89].

In contrast, the yeast histone mRNAs are polyadenylated but lack the 3’ stem-loop structure and SLBP protein. The 3’ UTR-dependent post-transcriptional regulation also contributes to their cell cycle expression pattern [90]. However, it is unknown what trans-acting factor that specifically binds the 3’ UTR. The yeast core histone mRNAs are degraded by exosome and Xrn1 with exosome degrading mRNA in 3’-5’ direction and Xrn1 degrading mRNA in 5’-3’ direction [91]. Xrn1 degradation pathway requires mRNA to be de-capped and the Lsm1-7-Pat1 complex stimulates de-capping of mRNAs. Deletion of exosome component *RRP6* results in continued accumulation of *HTB1* mRNA after S phase [92], while deletion of *LSM1* causes a G1-specific increase in histone mRNA levels [93]. It remains elusive how Lsm1-7-Pat1 was recruited to histone mRNA in budding yeast.

Histone expression is also regulated at translational and post-translational levels. In metazoans, SLBP coordinates gene translation by interacting with SLIP1, which interacts with the translation initiation factor eIF4G [94]. Whether core histone translation is subject to cell cycle regulation remains unclear. In budding yeast, a Rad53-dependent surveillance mechanism exists to regulate histone levels during normal cell cycle progression and in response to DNA damage [95]. Rad53 phosphorylates soluble histones that are not packaged into chromatin, leading to their ubiquitination and subsequent degradation by proteasome [95]. Mammalian proteasome PA200 appears to promote the selective loss of the core histones in elongated spermatids to prevent cell apoptosis [96].

Histone synthesis can also be regulated by amplification of histone genes. Libuda and Winston reported that the gene copy number of *HTA2*-*HTB2* increased by forming a new, small, circular chromosome when *HTA1*-*HTB1* was deleted [97]. This new circular chromosome was formed by recombination between two Ty1 retrotransposon elements flanking *HTA2*-*HTB2* locus [97]. This Ty1-mediated amplification allows histone genes to dosage-compensate in response to reduced histone levels [97]. In breast cancers, histone H2A *HIST2H2AC* expression was altered with 16.8% of the cases related to *HIST2H2AC* gene amplification and/or mRNA upregulation [98].

## REGULATION OF THE EXPRESSION OF HISTONE VARIANTS

In addition to the canonical histones, there are several variant histones whose synthesis is not cell cycle-regulated. The major histone variants are H3.3 and H2A.Z, which are found in all multicellular organisms. Other variants include the centromere-specific variant CENP-A (Cse4 in yeast), the testis-specific H3 variant H3t, and primate-specific variants H3.X and H3.Y [99, 100]. These histone variants are structurally similar to canonical histones but are distinct from their canonical counterparts. These histone variants play pivotal roles in modulating chromatin dynamics and chromatin-associated processes [101].

Histone variant H3.3 genes, namely *H3F3A* and *H3F3B* in humans, lie outside the histone gene clusters. In contrast to canonical histone genes that lack introns and their polyA tails are replaced with a special stem–loop structure, histone variants contain introns and their mRNAs have poly(A) tails and are processed like most other RNA polymerase II transcripts [102, 103]. The canonical histones are expressed and incorporated into chromatin at S phase, whereas histone variants are typically expressed throughout the cell cycle and is independent of DNA synthesis [101, 103].

## BIOLOGICAL SIGNIFICANCE OF HISTONE GENE REGULATION

The stability of chromatin structure is required for normal cell cycle progression. During DNA replication, the chromatin structure is disrupted ahead of DNA polymerase and parental histones are randomly distributed onto two DNA strands with 50% coverage [4]. To maintain the normal chromatin organization, cells need to synthesize a large amount of histones, providing ∼20 million new nucleosomes for packaging the newly replicated daughter strands [4]. Insufficient histone levels can trigger a cell-cycle arrest in budding yeast and impair S-phase progression in mammals [6, 23]. Reduced expression or depletion of core histones during DNA replication delayed the entry into S phase and makes cells suffer mitotic arrest [6, 104, 105].

Over-expression of histones and accumulation of soluble histones outside S phase triggers chromosome aggregation or loss and are toxic to cells [7]. Histones are degraded in response to DNA damage to enhance the chromatin dynamics and recombination rates and an accumulation of excess histones usually results in sensitivity to DNA-damaging chemicals or deleterious phenomenon [5, 106]. Elevated expression of *HTA1* and *HTA2* leads to a severe growth defect in budding yeast [53]. Spt21 degradation by APC/C^cdh1^ is required to ensure that histone gene expression is not activated during genotoxic stress [6]. Increased histone gene mRNA in *LSM* deletion mutant leads to genome instability and sensitivity to hydroxyurea, which causes S-phase arrest [93]. Mutation of the abnormal oocyte (ABO) gene, a protein that negatively regulates core histone gene expression in *Drosophila melanogaster*, is maternal-lethal during embryogenesis [107]. The mouse proteasome PA200 promotes the selective degradation of core histones (especially the acetylated histones) in elongated spermatids to prevent apoptosis and malformed spermatids in mouse testes [96]. Gunjan and Verreault showed that genotoxic agents that blocks replication could trigger rapid saturation of histone chaperones and the accumulation of free histones [95]. It is therefore likely that certain cancer chemotherapeutic drugs that interfere with DNA replication could lead to accumulation of free histones, which could be partly responsible for the cytotoxicity of these drugs.

Mis-regulated histone expression leads to aberrant gene transcription by altering the chromatin structure. Tightly packaged chromatin structure makes DNA less accessible for transcription machinery, whereas an open chromatin structure is prone to induce gene expression. It is conceivable that reduced histone expression leads to less well positioned nucleosomes and up-regulated gene expression, while an excess of histones blocks gene transcription. Indeed, decreased histone expression increases the expression of genes encoding enzymes of the tricarboxylic acid cycle and oxidative phosphorylation in budding yeast, which switches cell metabolism from glucose fermentation to oxidative phosphorylation [108]. Replicative aging is accompanied by reduced histone proteins, and this is a cause of aging in budding yeast [109]. During replicative aging, the nucleosome occupancy was decreased by 50% across the whole genome, leading to transcriptional induction of most yeast genes [109]. The aging-coupled histone loss also results in elevated levels of DNA strand breaks, mitochondrial DNA transfer to the nuclear genome, large-scale chromosomal alterations, translocations, and retrotransposition [109]. Increased histone supply during aging can efficiently increase the life span of budding yeast [110].

## HISTONE GENE REGULATION AND CANCER

Histone synthesis is intimately linked to DNA replication and this coupling could be used to identify cells with high replicative potential such as tumor cells [2]. Histone H2A type 2-C (*HIST2H2AC*) mRNA was increased in breast cancer cells and was necessary to induce proliferation in response to growth factors [98]. The reduction of *HIST2H2AC* mRNA levels induced the expression of cell cycle inhibitors, apoptosis effectors and possibly inhibition of mTORC1 activation by growth factors [98]. *HIST2H2AC* was expressed in primary breast cancer samples and analysis of The Cancer Genome Atlas (TCGA) provisional breast cancer data set (n=1098 patients) showed that 17% (189 cases) have a genetic alteration in *HIST2H2AC*, with 186 patients exhibit *HIST2H2AC* gene amplification and/or mRNA upregulation [98]. Increased *HIST2H2AC* expression was correlated to a higher proliferation [98]. In uterine and ovarian carcinosarcomas, the replication-dependent histone gene cluster *HIST1* and *HIST2* were amplified [111].

Many histone gene regulators are involved in cancer survival and proliferation. CBP/p300 is required to maintain the growth of castration-resistant prostate cancer [112, 113]. Inhibition of CBP/p300 bromodomain suppresses the growth of malignant melanoma, breast cancer, leukemia and prostate cancer [112, 113]. Some protein factors involved in histone gene regulation and nucleosome assembly could become potential biomarkers for tumor cells. For example, cyclin E2 is the major E-cyclin within HLBs in breast cancer cells and has a strong prognostic role in breast cancer [114, 115]. Cyclin E2 has a particular role in coordinating the cell cycle with histone transcription and can induce genomic instability that is associated with defects in chromosome condensation partly due to excessive histone production [114, 116]. Analysis of the transcriptome profiles of breast cancers from TCGA showed that high cyclin E2 expression is associated with high levels of replication-dependent histones, which could explain the correlations of high cyclin E2 expression with poor outcome and genomic instability in breast cancer [114]. The expression of ATAD2, the human homolog of Yta7 correlates with clinical outcome of breast cancer patients [57]. In addition, the chromatin assembly factor CAF-1 could become a convenient tool to discriminate proliferating and quiescent states due to its crucial role in DNA replication-coupled nucleosome assembly and S-phase progression [117]. CAF-1 was found to be over-expressed in breast cancer cells and its expression is positively correlated with the routinely used proliferation marker Ki-67 [117], implying the plausible application of CAF-1 as a powerful proliferation marker with potential prognostic value in breast cancer.

Some trans-acting regulators that control histone gene transcription have been reported to mutate in tumor cells. The class of genes encoding SWI/SNF is one of the most commonly mutated targets in cancer, which are collectively mutated in 20% of all human cancers [118]. Germline mutation of NPAT has been found in NLPHL (nodular lymphocyte predominant Hodgkin lymphoma) [119]. Exome sequencing revealed that in these patients there was a deletion of 3 bp resulting to the loss of serine 724 (S724) in NPAT [119]. The germline mutation of NPAT was found in several cases, implying it could function as a candidate risk factor for Hodgkin lymphoma [119].

## CONCLUSIONS

Histone gene expression is primarily regulated by a coordinated action of transcriptional factors, histone chaperones and chromatin-bound proteins [27]. Proper histone gene expression is important to maintain normal cell cycle progression, genome stability, DNA damage response and gene transcription. Although much progress has been made toward understanding the mechanism of histone gene regulation, many questions remain to be addressed. For example, how is HIR complex recruited to the NEG region? How does Rtt106 discriminate SWI/SNF and RSC and what are the cell cycle specific signal(s) trigger the switch? Although it is clear that Rtt109 and the putative HAT Spt10 are involved in histone gene regulation, their mechanisms of action remain to be determined. Much is known about regulation of histone gene pairs (*HTA1*-*HTB1*, *HHT1*-*HHF1 HHT2*-*HHF2*) containing CCR/NEG elements. Little is known about the mechanisms underlying regulation of the *HTA2*-*HTB2* gene pair that contains a histone UAS but not NEG. How is *HTA2*-*HTB2* regulated by a HIR-independent mechanism? How is the expression of all four core histones coordinated?

Proper histone expression is required for tumorigenesis. Elucidating histone regulation mechanism will shed lights on understanding the side effects of cancer chemotherapy and development of cancer prognostic biomarkers. Certain cancer chemotherapeutic drugs could lead to accumulation of free histones and have side effects. Many regulators including CBP/p300, ATAD2, Cyclin E2, SWI/SNF and NPAT play important roles in cancer survival and proliferation. Some trans-acting regulators that control histone gene transcription have been reported to harbor mutations in tumor cells. Yet, it remains unclear whether these mutations or dysfunctions contribute to tumorigenesis by altering histone synthesis. Investigating this probability will undoubtedly provide novel insights into their roles in tumorigenesis.
